# Confounding Effect of Wetting, Compaction, and Fouling in an Ultra-Low-Pressure Membrane Filtration: A Review

**DOI:** 10.3390/polym14102073

**Published:** 2022-05-19

**Authors:** Tok Sheng Hung, Muhammad Roil Bilad, Norazanita Shamsuddin, Hazwani Suhaimi, Noor Maizura Ismail, Juhana Jaafar, Ahmad Fauzi Ismail

**Affiliations:** 1 Faculty of Integrated Technologies, Universiti Brunei Darussalam, Gadong, Bandar Seri Begawan BE1410, Brunei; 18b4016@ubd.edu.bn (T.S.H.); roil.bilad@ubd.edu.bn (M.R.B.); hazwani.suhaimi@ubd.edu.bn (H.S.); 2 Faculty of Engineering, Universiti Malaysia Sabah, Jln UMS, Kota Kinabalu 88400, Malaysia; 3 Advanced Membrane Technology Research Centre (AMTEC), N29A, Universiti Teknologi Malaysia, Johor Bahru 81310, Malaysia; juhana@petroleum.utm.my (J.J.); afauzi@utm.my (A.F.I.)

**Keywords:** ultra-low-pressure filtration, gravity-driven membrane filtration, membrane fouling, membrane compaction, membrane wetting

## Abstract

Ultra-low-pressure membrane (ULPM) filtration has emerged as a promising decentralized water and wastewater treatment method. It has been proven effective in long-term filtration under stable flux without requiring physical or chemical cleaning, despite operating at considerably lower flux. The use of ultra-low pressure, often simply by hydrostatic force (often called gravity-driven membrane (GDM) filtration), makes it fall into the uncharted territory of common pressure-driven membrane filtration. The applied polymeric membrane is sensitive to compaction, wetting, and fouling. This paper reviews recent studies on membrane compaction, wetting, and fouling. The scope of this review includes studies on those phenomena in the ULPM and how they affect the overall performance of the system. The performance of GDM systems for water and wastewater treatment is also evaluated. Finally, perspectives on the future research direction of ULPM filtration are also detailed.

## 1. Introduction

Clean water is increasingly restricted internationally due to population increase, climate change, and pollution. In 2018, 663 million people did not have access to clean water [1]. Many live in remote areas with poor access to the water distribution grid. Ultra-low-pressure membrane (ULPM) filtration could help pave the way for universal access to clean water due to its ability to operate without physical and chemical cleaning. Much research has been conducted on different feeds, including diluted wastewater and surface water with varying amounts of pollutants, which seems promising [2]. The system operates at ultra-low pressures (ULPs) of 0.04–0.06 bar, in comparison to conventional ultrafiltration (UF), which operates at trans membrane pressures of 3–5 bar and consumes much less energy [3,4]. Without backwashing or routine cleaning, stable fluxes of 2–20 Lm^−2^h^−1^ were achieved in a lab-scale test [5,6], demonstrating a maintenance-free system. The process is often operated without the need for electrical energy. The filtration is solely driven by gravity, so the common term coined for this process is gravity-driven membrane (GDM) filtration. GDM may be utilized to conserve energy in advanced wastewater treatment and domestic water treatment. However, as with any other membrane filtration, it is subject to the effects of fouling, wetting, and compaction.

Particles, partly soluble organic and/or inorganic macromolecules, and/or biological microorganisms will inevitably be deposited on the membrane surface [7]. When foulants are deposited on the membrane’s surface or inside its pores, they reduce permeate flow, alter selectivity and permeability, and decrease the membrane’s lifespan when chemical cleaning is necessary to restore performance. Pore blockage results in cake layer formation, dominated by inorganics or natural organic matter molecules, depending on the feed and membrane pore size. Following initial colonization of the bacterial membrane surface by bacteria, the development of biofilms will also increase with increasing pressure. Furthermore, biofilm increases membrane resistance, leading to a decrease in ULPM flux [8]. On the other hand, biofilms have been known to act as a second “membrane”, improving the removal efficiency of ULPM. Biofilms have also been attributed to flux stabilization during long-term usage of ULPM, and flux is proportional to the thickness and structure of the biofouling layer [9].

Membrane compaction is a well-known phenomenon in pressure-driven membrane processes. The pressure on the feed side compresses the membrane during high transmembrane pressure, changing the membrane’s structure and permeability [10]. Due to mechanical deformation, compressive strain results in a decrease in membrane thickness. Compaction can be divided into either irreversible or reversible. When the applied pressure is removed, the thickness of the membrane may increase quickly and time-dependently, which is the recovery process. Even when left unloaded for extended periods, the membrane often maintains some deformation during recovery [11], which signifies irreversible compaction.

For UF with moderate pressure (2–5 bar), it has been reported that compaction did have an adverse effect on the filtration capability of the membrane [12,13,14,15]. In addition, separation efficiency has also been shown to be affected and enhance molecule retention by lowering the size of the membrane pore or deforming the structure of the pore, which will also increase the tendency of fouling [16,17,18,19]. However, Tarnawski and Jelen [18] showed that compaction decreased the permeability of a polysulfone membrane without altering the selectivity.

Most membranes used for liquid and gas separations are made from polymer materials. ULPM filtration is an emerging process employing very low transmembrane pressure and is designed for decentralized system applications. The compaction phenomenon in traditional pressure-driven filtration is not dominant due to high transmembrane pressure. However, it is highly important in ULPM filtration, particularly when treating feeds with low membrane fouling propensity.

It has been shown that membrane wetting increases the flux [20,21]. The wetting condition of microfiltration (MF) and UF membranes significantly affects their performance [21,22,23]. Even after considerable filtration, certain membranes, particularly hydrophobic membranes in microporous sections, remain largely dry [21,22]. At a flux of 200 Lm^−2^h^−1^bar^−1^, complete wetting took at least six hours. Due to the non-uniform pore size distribution in polymer networks produced during fabrication (mainly through the phase inversion process), incomplete wetting occurs. During wetting, pores of varying sizes moistened and linked by a fluid create communication networks that result in non-uniform wetting [16] and undesirable effects such as fluid droplet entrapment and flow bypass [23]. Additionally, a membrane that is insufficiently wetted inhibits flux in two ways: (1) the overall flux falls linearly as the number of unwetted holes increases, and (2) a membrane that is not uniformly wetted permits liquid to flow through it in a variety of ways. This modifies the flow patterns inside the membrane, resulting in unfavorable channels that contribute to a reduction in liquid mass transfer [24].

The progress of GDM developments has been reported extensively [25]. Membrane fouling has been overwhelmingly acknowledged in ULPM filtration but less so for the others. Compaction, membrane wetting, and fouling occur simultaneously during filtration, much more so at very low transmembrane pressure (TMP). Unfortunately, due to its complexity, membrane compaction, wetting, and fouling are seldom discussed in ULPM process studies. Process engineers often confuse occurrences of compaction and wetting properties by lumping them together as membrane fouling. Realizing the increasing importance of ULPM processes, it is essential to understand the compounding effects of those three phenomena, which are discussed critically in this review. Additionally, investigating membrane fouling in ULPM filtration will improve the system’s implementation.

## 2. Ultra-Low-Pressure Membrane Filtration

The term “ultra-low pressure” in the ULPMF system is defined as a TMP of <0.1 bar. The new terminology is important to distinguish it from the common MF and UF, despite using similar membrane types. Such a low pressure is normally obtained from the feed hydrostatic pressure from the water head of <1.00 m. This definition excludes the submerged membrane bioreactors (MBRs) that typically also work under low TMP. A number of studies have explored hydrostatic pressure as the driving force of filtration, often called GDM filtration. However, due to the expansion of the concepts to other applications and the possibility of imposing pressure by different means, the term ultra-low pressure is proposed in this review.

**Figure 1:**
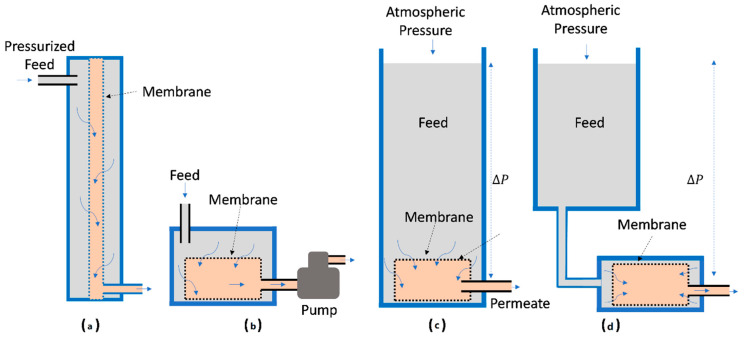
Basic illustrations of ultra-low-pressure membrane filtration system driven by mechanical and hydrostatic pressure showing (**a**) pre-pressured feed, (**b**) vacuum system involving permeate pump and gravity-driven with (**c**) internal and (**d**) external membrane placements.

ULPM filtration can work under various basic configurations, as illustrated in Figure 1. The system consists of a feed tank where the membrane can be submerged and linked externally. The system operates under a constant hydrostatic pressure that can be adjusted by changing the level of feed or constant flux with a permeate pump as long as the TMP is less than the defined threshold. The TMP can also be generated by pumping the feed to generate positive pressure or suction from the permeate side to generate negative pressure to drive the filtration.

## 3. Membrane Compaction

Membrane compaction refers to the change in the physical structure of the membrane material due to exposure to the TMP. Due to the flexible nature of the polymer within the matrix, it affects the hydraulic filtration performance. For traditional pressure-driven filtration (MF, UF, nanofiltration (NF), and reverse osmosis (RO)), compaction is mostly insignificant because of the high applied TMP (2–70 bar). However, it becomes important when the TMP is very low, such as in ULPM filtration employing polymeric membranes. In a recent study on ULPM filtration under a GDM system employing a hollow fiber membrane, the clean water permeability decreased from 720 to 500 to 426 Lm^−2^h^−1^bar^−1^ when ΔP was increased from 2.2 to 3.2 to 10.0 kPa [26]. The impact of compaction was also seen when the system was used for activated sludge filtration. More than 50% of clean water permeability loss was observed when the TMP was increased from 1 to 10 kPa in a ULPM filtration system employing a hollow fiber operated under a constant-pressure system (our unpublished work).

Similarly, for a flat-sheet membrane, the permeability decreased from 2740 Lm^−2^h^−1^bar^−1^ to 376 Lm^−2^h^−1^bar^−1^ when the TMP was increased from 2.5 to 19.0 kPa [10]. Those findings categorically show the importance of polymeric membrane compaction in ULPM filtration. As such, a detailed discussion on this aspect is required.

### 3.1. Measurement

Off-line examinations of membrane compaction (such as scanning electron microscopy (SEM) analysis or micrometer measurements) cannot determine how compaction affects filtration capacity. The impact of compaction may also be observed when pure water permeability measurements are made before and after filtration of a process stream. However, the information provided by such measurements does not always indicate the effect of compaction but also that of membrane fouling [15].

Membrane compaction has also been assessed using data from various mechanical testing configurations [12,27]. However, the conditions used in these tests were much different from those found in a filter cell during filtering. As a result, the data lack accuracy regarding membrane compaction during filtration and its influence on filtration performance.

Real-time measurements offer the most accurate information on compaction during filtering under specific situations. Peterson et al. [28] established ultrasonic time-domain reflectometry (UTDR) for real-time monitoring of reverse osmosis and Zirfon composite membrane compaction. Reinsch et al. [29] showed the use of UTDR to monitor gas separation membrane compaction. Their findings demonstrate that UTDR is a realistic non-invasive technology that can effectively monitor membrane compaction in real time. As a result, the primary shortcoming of off-line methods is that they can demonstrate only irreversible compaction.

A significant limitation of prior compaction research is the difficulty in obtaining simultaneous real-time measurements of permeate flow and membrane thickness changes. As a result, compressive stresses have been determined indirectly using flux-decline tests or independently via several mechanical testing configurations. While the former is accurate depending on the assumptions underlying the flux-decline model used, compressive strains measured via the latter typically overestimate the actual mechanical response because the influence of fluid under pressure flowing through the membrane pores is not included in these “static” tests.

### 3.2. Permeability

**Figure 2:**
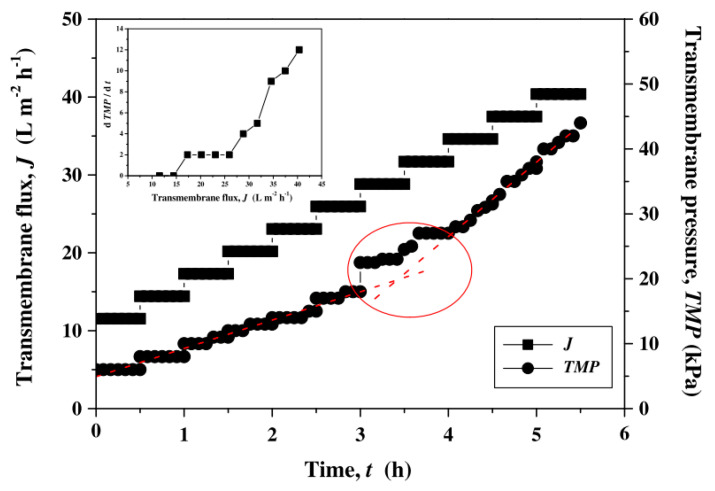
The transmembrane flux (J) and pressure (TMP) as a function of time (t) for seawater filtration experiments with increasing flux steps. Reprinted with permission from Ref. [26]. Copyright 2011 Elsevier.

Compaction happens when the membrane structure is compressed due to the TMP. It generally decreases the membrane permeability due to the deformation of the polymer matrix, pore constriction, and other phenomena. Di Profio et al. [30] demonstrated that as pressure was raised on polyethersulfone hollow fiber membranes, the more porous support layer densified, resulting in the thickening of the skin layer (selective barrier). As a result, thicker membranes had a lower permeability, and the permeability decreased gradually throughout the compaction period, as indicated in Figure 2.

For hydraulic pressure-driven membrane processes, densification of the skin layer also leads to a gradual increase in the mass transfer resistance and hence a decrease in membrane permeability over time. Membrane compaction can also reduce the water permeability coefficient of the membrane during the RO process, with the effect being more noticeable at higher pressures and temperatures [31]. Additionally, other studies show that membrane compaction may result in a considerable increase in the resistance of the porous support layer of thin-film composite (TFC) membranes, which may contribute considerably to the membranes’ overall resistance [32].

Additionally, it is worth noting that the relationship between porosity and permeability is linear, meaning that permeability is directly proportional to flux. Permeability is proportional to the membrane’s volume porosity [33]. Thus, a reduction in permeability is seen as a reduction in membrane porosity. Reduced membrane pore size or pore geometry compaction can also improve the retention of molecules with a magnitude equal to or less than the molecular weight cut-off (MWCO) value [15].

Persson et al. [33] evaluated the permeability and thickness of polyamide, polysulfone, and cellulose acetate UF membranes following static compression in a hydraulic press. Compression of the membrane led to a significant decrease in its thickness at a TMP of just 300 kPa. Additionally, compacting the polysulfone membrane decreased its permeability by 60%, but only 35% for the cellulose acetate membrane. They postulated that the disparate compaction effects were caused by differences in membrane structure, with the polysulfone membrane’s high degree of compaction caused by the deformation of the macrovoids in the membrane substructure.

It is generally anticipated that a membrane component with high porosity, such as the support layer, will deform more severely than other membranes [34,35,36,37,38]. As a result, displacement of the support layer may greatly contribute to the decreases in TFC water permeability caused by compaction [34,39,40]. Additionally, deformation between the support and selective layers at the interface diminishes the effective membrane surface area, contributing significantly to the permeability reduction [41]. While it is usually thought that the water permeability coefficient is constant, variations are regularly seen and are most frequently attributed to compaction, fouling, or a mix of the two [42].

**Figure 3:**
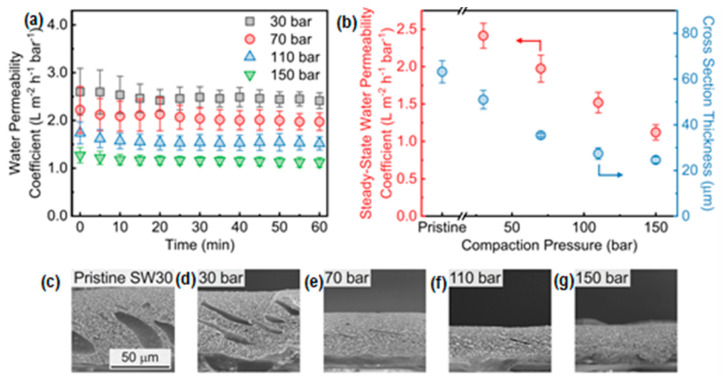
Characterization of commercial SW30 thin-film composite membranes following compaction at hydraulic pressures up to 150 bar (**a**–**g**). Reprinted with permission from Ref. [43]. Copyright 2020 Elsevier.

According to Davenport et al. [43], most compaction effects occur immediately upon applying pressure, with very little time-dependent compaction identified during 12 h (Figure 3). The water permeability coefficient reaches a steady state in less than an hour, and there is a substantial negative correlation between the water permeability coefficient and increasing hydraulic pressure. The steady-state water permeability coefficient decreases by 35% as compaction increases, from 2.0 Lm^−2^h^−1^bar^−1^ at 70 bars to 1.3 Lm^−2^h^−1^bar^−1^ at 150 bar. At high applied pressures, water permeability decreases, resulting in reduced flux and greater energy consumption in conventional RO and high-pressure RO processes.

Compaction had the greatest morphological effect on the support layer, resulting in a 60% drop in cross-sectional thickness under a TMP of 150 bars. Additionally, it was demonstrated that compaction could reduce the surface porosity of the support layer by up to 95%. Although this decrease in porosity was identified as the cause of the compaction-induced decrease in water permeability, the selective layer’s intrinsic permeability remained unchanged by the compaction. Due to the reduction in water permeability induced by compaction, it is essential to use higher operating pressures and spend more energy to maintain a constant water flow and plant capacity [43].

In contrast to these studies, which all report a decrease in permeability (or an increase in resistance) as pressure increases, Lawson et al. [27] used gas permeation data to demonstrate that compressing microporous polypropylene membranes for 5 min at 0.002 bar increased membrane permeability by up to 11%. Although this study contradicts past findings in similar domains (such as RO and UF), microporous membranes function at a substantially lower TMP. They are more porous than RO and UF membranes. Under these circumstances, the advantages of shortening the path across the membrane (which tends to increase permeability) balance the disadvantages of decreasing membrane porosity and pore size (which tend to decrease permeability).

**Figure 4:**
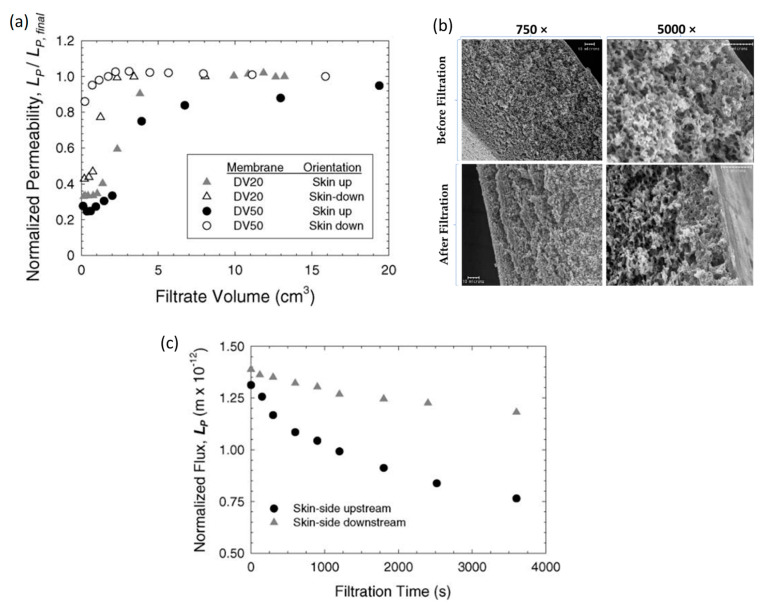
(**a**) Normalized permeability as a function of filtrate volume for the DV20 and DV50 membranes at a constant pressure of 155 kPa (22.5 psi) through a 30 kDa inline filter. (**b**) Scanning electron micrograph showing the Virsolve 180 membrane after filtration in the skin-side up orientation at 103 kPa for 30 min. (**c**) Normalized flux as a function of time for the Viresolve 180 membrane in the skin-side up and skin-side down orientations at a constant pressure of 103 kPa (15 psi). The PBS was pre-filtered through a 0.2 μm pore size membrane. Reprinted with permission from Ref. [44]. Copyright 2015 Elsevier.

Furthermore, Bohonak et al. [44] showed that when DV20, DV50, or Omega 300 membranes were exposed to a TMP of 1.55 bar for more than 2 h, there was no trace of compaction (Figure 4a). Conversely, when the pores became fully saturated, the permeability of these membranes increased, with the DV-series membranes having the greatest effect. SEM micrographs demonstrated that the structure of the Viresolve 180 membrane remained unchanged (Figure 4b). However, this might just be a result of the membrane’s “decompression” during drying and sample preparation, along with the low resolution of the scanning electron micrographs. However, Figure 4c demonstrates that after 30 min, the filtrate flow through the Viresolve 180 membrane operated skin-down dropped by 20% due to membrane compaction. This compaction behavior exhibited reversible and irreversible components, with minimal compaction identified at pressures as low as 6 kPa. This time-dependent compaction was found only when the membrane was oriented skin-side down, which might be explained by the deformation of the support layer behind the skin layer in this orientation.

### 3.3. Effect of Transmembrane Pressure

Although compression investigations with MF and UF membranes are limited, there is compelling evidence that these larger-pore membranes can be compressed at low pressures. For example, Bowen and Gan [45] observed a 25% drop in flow for MF membranes after only 40 min of compression at 200 kPa. Tarnawski and Jelen [18] showed that UF membranes operated under TMP pressures significantly lower than 400 kPa exhibited significant compaction, with compaction accounting for the bulk of the flow reduction seen during cottage cheese whey UF.

A significant drop in permeability occurred during the first operation of membranes due to membrane structural compaction [39] because compaction happens due to the membrane’s polymer matrix becoming distorted. As the membrane matrix becomes compressed and finds equilibrium, the permeate flow slows and the rejection increases. Typically, the flux reduction is significant, reaching up to 60% of the initial flux [46,47].

Both porosity and effective route length may decrease during compaction. The relative changes in both values, on the other hand, are structure-dependent. For a structure with low porosity, such as the polyamide active layer, it is expected that the flux will decline linearly with thickness as the porosity drops (changing substantially faster) [36]. On the other hand, very porous structures boost flow due to the decreased effective route length.

Tarnawski and Jelen [18] discovered that GR60P polysulfone UF membranes were approximately 40% compacted under a TMP of 0-1MPa. With applied pressure, the membrane coefficient decreased from 7.2 × 10^−14^ to 4 × 10^−14^ m, and the decline was exponential (33% loss over the TMP range 0–0.5 MPa). Despite the compression of the membranes, the selectivity remained nearly constant. It was verified by demonstrating that the flow declined while the selectivity of asymmetric cellulose acetate membranes remained consistent over time [11]. The flux loss was attributed to the porous sublayer compacting, whereas the skin remained unaffected. Membrane compaction has been observed to impair membrane performance and efficiency in all trials reported elsewhere [15].

Additionally, it should be stressed that the loss of permeability caused by compaction is somewhat compensated for in a hydraulic scenario by increasing the number of permeable pores. This effect should be absent if the membrane is saturated, as no new liquid passes through the pore. According to Persson et al. [33], the order of permeabilities was as follows: cellulose acetate > polysulfone > polyamide. Cellulose acetate had a lower glass transition temperature (80 °C) than polysulfone (190 °C). The difference in permeability loss appears to be unrelated, as cellulose acetate should be more pressure-sensitive than polysulfone in this circumstance. Instead, the drop in permeability is mostly due to the membrane’s porosity and viscoelastic properties. The polysulfone membrane is more porous than the cellulose acetate membrane in volume. The PS membrane has a higher viscosity than the cellulose acetate membrane.

Additionally, this is supported by other publications. Tessaro and Jonsson [11] revealed that a PSU UF membrane lost part of its filtration ability when subjected to a continuous transmembrane pressure of 1.8 bar. When PSU and cellulose acetate UF membranes were used at 0–3 bar pressures, Persson et al. [33] reported compaction and reduced filtration performance.

Hitsov et al. [40] showed the first point in the SEM measurement of an uncompressed flat-sheet membrane sample at 92 µm prior to its use in full-scale direct contact membrane distillation. The membrane was tremendously compressed up to a TMP of 1.5 bar to a final thickness of 50 µm. This effect cannot be disregarded since the membrane was compacted by 30% within the module’s operating pressure range of up to merely 300 mbar.

Persson et al. [33] discovered that at 1 bar, the thickness of the UF membranes examined decreased by less than 10%. Zhang et al. [48] reported equivalent compaction as a function of pressure for a stretched polytetrafluoroethylene membrane, with a 22% thickness loss at 0.3 bar. However, the phase-inverted hollow membrane was almost incompressible across the 0.3 to 0.7 bar pressure range. These findings demonstrate that stretching can result in mechanically weak membranes susceptible to compaction, such as the stretched polytetrafluoroethylene and polyethylene membrane investigated by Zhang et al. [48].

### 3.4. Reversibility of Membrane Compaction

Persson et al. [33] compressed flat-sheet UF membranes for at least one hour at pressures ranging from 0.1 to 0.6 MPa and discovered that the flux was never regained following the compaction. The flux reduction increased when the precompression pressure was increased. This might be because the pressure exceeded the point of reversible compaction. On the other hand, other tests revealed that reversible compression is possible at low pressure. Compression of RO membranes may also be partially reversible, as evidenced by the increase in the water permeability coefficient when pressure decreases [49,50].

**Figure 5:**
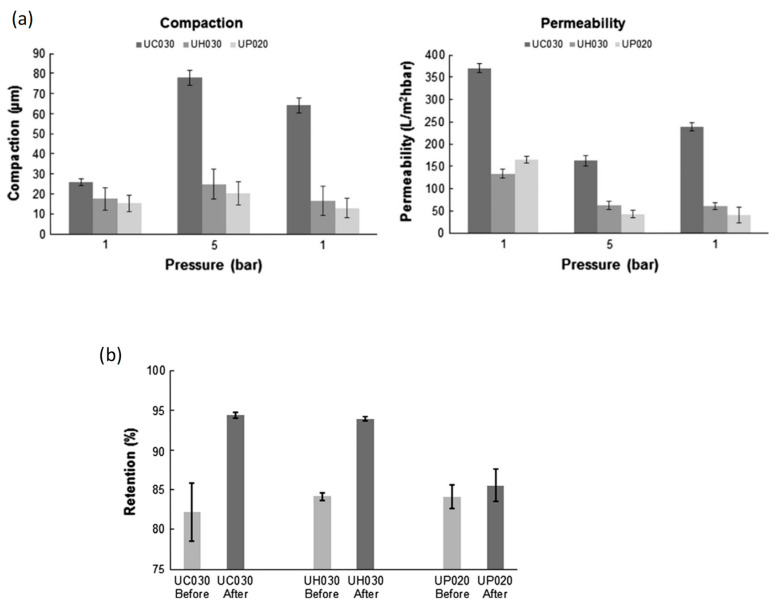
(**a**) Compaction and permeability of UC030, UH030, and UP020 membranes when compacted for 2.5 h at 5 bar; (**b**) effect of compaction (15 h at 7 bar, 30 °C) on the membrane retention of 8 kDa polyethylene glycol (UH030 and UC030 membranes) and 6 kDa polyethylene glycol (UP020 membrane). Retentions were measured at a flux of 100 Lm^−2^h^−1^, and the average results from three different membrane sheets are shown. Reprinted with permission from Ref. [15]. Copyright 2013 Elsevier.

Stade et al. [15] showed that the bulk of the compaction was observed between 1 and 7 bars in UH030 (polyethylene and polypropylene) and UP020 (polyethersulfone) membranes, but the compaction was reversible. After a pressure reduction from 5 to 1 bar, UC030 regained 40% of its original permeability (Figure 5a). Compaction at 7 bars enhanced the retention of polyethylene glycol (8 kDa) from 81 to 94 percent for UC030 and UH030 membranes, respectively, and from 83 to 93% for UH030 membranes. Retention values of >90% showed that the MWCO had changed to less than 8000 g/mol following compaction.

Polyethersulfone membranes preserved some permeability but not considerably. The membranes’ retention of polyethylene glycol molecules was seen before and after 15 h of compaction at 7 bar (Figure 5b). The UC030 membrane showed the largest retention improvements: after compaction at 7 bars, its polyethylene glycol (8 kDa) retention was approximately 14% higher (94%) than the value recorded before compaction. The UH030 membrane’s polyethylene glycol (8 kDa) retention increased by 10% following compaction.

**Figure 6:**
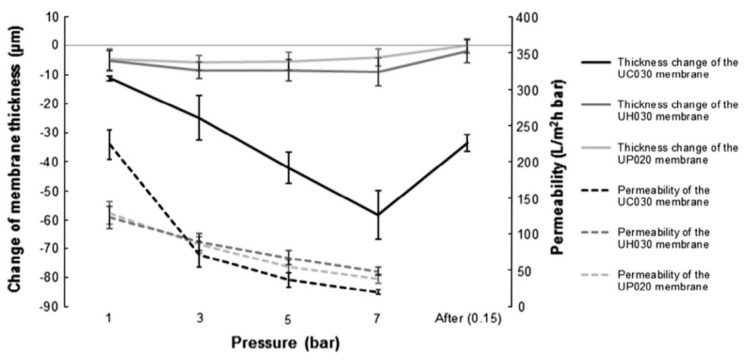
Compaction and permeability of UC030, UH030, and UP020 membranes at different pressures. Reprinted with permission from Ref. [15]. Copyright 2013 Elsevier.

Although the permeability of pure water decreased dramatically with increasing pressure, neither the drop in permeability nor the membrane thickness altered proportionately, as shown in Figure 6. The decreased permeability of pure water might result from the increased hydrodynamic resistance produced by the skin layer compaction. The polyethersulfone membranes UH030 and UP020 almost fully recovered from the modest compaction encountered during the recovery studies. However, their permeability remained unchanged (Figure 5a). The majority of the compaction of the UC030 membrane appeared to take place underneath the rather thin skin layer. This might account for a portion of the membrane’s increased hydrodynamic resistance, perceived as decreased permeability. Notably, the permeability decrease caused by reversible compaction may be confused with concentration polarization. Reversible compaction accounted for approximately 40% and 30% of the total compaction, respectively, when the UC030 membrane was operated at 7 or 5 bar (Figure 5 and Figure 6).

Peterson et al. [28] reported a similar effect when they utilized ultrasonic time-domain reflectometry to monitor the compressive strain of a reverse osmosis membrane: the water permeability continued to decline after the compressive strain reached stable values. Additionally, they speculated that the cause might be the skin layer’s densification. They were, however, unable to substantiate this claim due to the limits of their ultrasonic time-domain reflectometry technology.

## 4. Membrane Wetting

At the beginning of filtration with a virgin membrane, not all pores are wetted. Smaller pores are excluded from filtration because the cohesive forces between water molecules are higher than the adhesive forces at the liquid–solid interface. In other words, the liquid–solid surface tension is less than the liquid–air surface tension. Following filtration at a higher TMP, the smaller pores are also wetted, which contributes to the effectiveness of the membrane filtration system [21]. Thus, wetting, pore activation, and contaminant suppression play a critical role in achieving maximal water flux [19].

The absolute permeability value is proportional to the number of active pores used in the filtering process and increases at higher TMP. Substances with low surface tension such as acetone, isopropyl alcohol, and ethanol contribute significantly to the activation of smaller pores by lowering the membrane’s hydrophobicity via decreased surface tension. It draws water due to its hydrophilic characteristics while sticking to a hydrophobic non-polar surface.

Generally, a hydrophilic membrane possesses greater permeability. However, Xu et al. [37] suggested that membrane pores with a high hydrophobicity would exhibit ultra-high water permeability due to their low friction and the absence of polymer swelling. Experimental evidence suggests that membranes used in various applications, ranging from MF to RO, should exhibit a high degree of hydrophilicity to provide adequate water permeability [51,52,53]. On the other hand, molecular dynamic simulations show that hydrophobic holes promote increased water flow [54,55].

Hydrophilicity enhances the interaction of water molecules with pore walls and has an opposing effect on water permeability. On the plus side, the hydrophilic contact may boost the capillary force of the water infiltration, assisting the membrane in absorbing water molecules and increasing its wettability. With decreasing pore diameters, the capillary force of infiltration increases considerably. When the membrane’s pore size is reduced to the sub-nanometer range, water molecules inside the membrane have a larger chance of forming hydrogen-linked networks, which is critical for water transport in restricted spaces [56]. On the other hand, higher friction between water molecules and pore walls decreases flow velocity [52]. As the pore size decreases, a greater fraction of water molecules will contact the pore wall, amplifying the friction effect.

Xu et al. [36] demonstrated that a high hydrophobicity led to an increase in water permeability, as well as a high threshold pressure drop (Δ𝑃_T_). It was only when the applied pressure drop (Δ𝑃) surpassed the Δ𝑃_T_ that a high permeability occurred. For hydrophobic membranes, Δ𝑃_T_ values are typically at the scale of several hundreds of MPa, far more than experimentally accessible pressure drops. Δ𝑃 is typically set to an exceptionally high value obtained from simulations. This high Δ𝑃 value might easily exceed Δ𝑃_T_, maintaining the membranes in a moist condition at all times. In contrast to hydrophobic membranes, hydrophilic membranes might be wetted at low Δ𝑃, showing that Δ𝑃_T_ is close to zero. This would account for the discrepancies between experimental and simulated results.

Another reason for the increase in permeability caused by the wetting agent is the swelling of the membrane skin layer, as demonstrated by changes in thickness measurements and the consideration of polymer solubility parameters, indicating a degree of polymer plasticization, as well as the enhanced removal of membrane preservatives and polyvinylpyrrolidone, a common pore-forming agent. This was demonstrated in situ by pretreating commercially available polysulfone UF membranes with ethanol, resulting in a threefold increase in the obtained clean water permeability values [22]. While initial and continuous ethanol treatments boost initial flux, they may have a detrimental effect on the membrane’s performance and longevity over numerous cycles. It was discovered that the elastic modulus was lowered owing to polyvinylpyrrolidone elimination and plasticization.

In the case of porous membranes, wettability is influenced by three factors: the pore size, the liquid’s surface tension, and the membrane material’s surface energy. The alcohol wetting of a hydrophobic membrane resulted in a considerable decrease in membrane resistance at alcohol concentrations greater than 30% wt [21].

The permeability of hollow fiber polyethersulfone membranes changed over time when filtration experiments were conducted at permeate fluxes of 15, 30, 15, 50, and 15 Lm^−2^h^−1^ [21]. The wettability of the membrane explains this phenomenon. The experiment was designed to compare the permeability of virgin and moist membranes. In each of these circumstances, the permeability was significantly enhanced. The permeability of polysulfone flat-sheet membranes quadrupled. The rise was less pronounced with the polyethersulfone hollow fiber membranes. Their permeability increased from around 1000 to nearly 2000 Lm^−2^h^−1^bar^−1^. Sludge supernatant was employed as a feed in one of the experiments. The permeability of the sludge supernatant decreased by approximately 50% during the first 100 s of the filtration. Finally, the fluxes of wetted membranes stabilized at 86 Lm^−2^h^−1^bar^−1^ for ethanol and 82 Lm^−2^h^−1^bar^−1^ for isopropyl alcohol, which are much greater than those of a virgin membrane, which stabilized at 58 Lm^−2^h^−1^bar^−1^, demonstrating the benefits of wetting the membrane.

## 5. Membrane Fouling

In conventional MF/UF membrane processes with effective fouling control strategies, an increased driving pressure generally leads to an almost linear increase in the permeate flux. However, as demonstrated in multiple studies [57,58,59,60], changing the TMP did not substantially change the flux in the ULPM filtration system. This indicates that when pressure increased during membrane filtration, the overall resistance of the fouled membrane increased. This impact has been linked to the fouling layer being compressed at higher pressures, resulting in decreased porosity and increased fouling layer resistance [60].

Tang et al. [61] tested the ULPM filtration system for reservoir water treatment at three different operating pressures (60, 120, and 200 mbar). At a TMP of 200 mbar, the flux stabilized at roughly 8.6 Lm^−2^h^−1^, which was only slightly higher than the steady flux of 6.6 Lm^−2^h^−1^ at the lowest pressure (60 mbar). As a result of reduced energy consumption, it is indicated that lower pressure (40–60 mbar) is more appropriate for this sort of passive membrane filtration process.

### 5.1. Flux Stabilization

Over the last decade, GDM filtration has been researched. The technology is defined not only by a comparatively low TMP, which can be obtained simply using gravity and hence operated with extremely low energy consumption, but also by the flow stabilization phenomenon. In GDM, microorganisms, organic aggregated colloidal material, and particulate organic and inorganic material can be rejected by the membrane and subsequently accumulate on the membrane surface during GDM filtration. These trapped substances tend to create a biofilm layer on the membrane, referred to as a “mini ecological system.”

The biofilm formed on the membrane increases dissolved organic substances by acting as a secondary membrane (separation function) or performing organic substance biodegradation. This enables consistent operation for up to a year without cleaning or flushing. It was demonstrated that a dead-end operated UF system operating without chemical or physical hydraulic fouling and biofouling management resulted in a steady flux value over a long time, with a biologically active fouling layer playing a critical role in governing the flux stability. In laboratory investigations, stable flow values were obtained for months of operation and one year in field tests [51]. Additionally, Peter-Varbanets et al. [2] investigated flux stability in a side-stream GDM system. They determined that the steady flow was induced by the deposition and production of non-soluble material and irremovable fouling and fouling structural changes caused by biological activities.

### 5.2. Effect of Feed on the Stable Flux

GDM systems have been used in decentralized potable water treatment, decentralized non-potable water treatment, wastewater treatment, and pretreatment of saltwater for desalination. In general, feed water with a greater concentration of organic compounds forms a more resistant biofilm, resulting in permeate flux in the following sequence: (diluted) wastewater/greywater rainfall/ river water/seawater. This tendency was reinforced by the following facts: (1) adding wastewater to river water resulted in a decreased steady flux value; (2) the average flow was 5 Lm^−2^h^−1^ while treating low-organics lake water but reduced to 2 Lm^−2^h^−1^ when treating high-organics lake water (owing to algal development) [53].

This finding is further corroborated by Kunzle et al. [54] and Ding et al. [55], who demonstrated that the comparatively low flow in the case of greywater is related to the water’s high overall fouling potential. Pretreatment of the feedwater with granulated activated carbon adsorption increased the steady flow in GDM from 2 to 6 Lm^−2^h^−1^ [51]. This impact was attributed to the elimination of foulants and the enhancement of the environment for higher organisms to flourish. While the study described above was conducted on river water, the same technique might be used for greywater.

Perter-Varbanets et al. [2] conducted a filtration test using feed water with various total organic carbon (TOC) contents taken from a river, a lake, and diluted wastewater. They discovered that diluted wastewater with a TOC of 12.5 mg/L had the lowest flux level, stabilizing at around 7 Lm^−2^h^−1^, and river water with a TOC of just 2.5 mg/L had the greatest flux, reaching up to 10 Lm^−2^h^−1^. It was determined that feed water with a greater TOC content resulted in increased fouling development on the membranes, resulting in a decreased flux throughout the long-term operation.

Perhaps, more organic compounds in the feed water result in increased organic buildup in the biofilm matrix. This might be because the organic quantities deposited on the membrane surface were adequate and beyond the capacity of microbes to use them or because GDM systems have low oxygen levels. Such oxygen levels may be detrimental to the development and predation activities of eukaryotes isolated from the feed water.

### 5.3. Mechanism of Flux Stabilization

Membrane fouling is a hot topic of research in GDM. According to recent research, the early stage of GDM filtration is dominated by pore blockage. In contrast, the steady flow is attributable to the cake layer building on the membrane surface throughout the expanded operating duration [49].

Wang et al. [62] conducted a GDM experiment with two feeds that had varied fouling potential TOC concentrations of 4.9 ± 0.2 mg/L (E1) and 2.6 ± 0.1 mg/L (E2). They classified the fouling process into four distinct stages based on its behavioral and trend characteristics. Additionally, membrane fouling resulted in time-dependent flux variations during membrane filtration, which may be categorized physically into three stages: (a) initial foulant adsorption and deposition (phase 1), (b) pore constriction and obstruction (phases 2 and 3), and (c) cake or gel layer development (phase 4).

**Figure 7:**
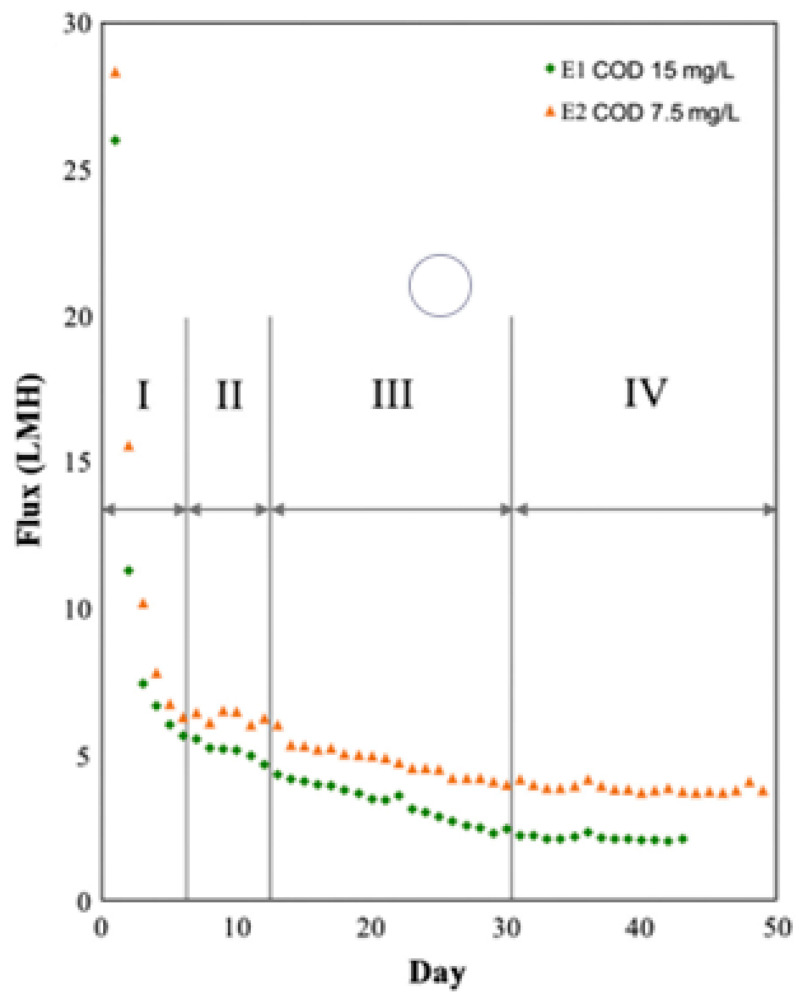
Variations in flux and resistance during the experiments were divided into four phases: flux (initial E1 and E2 were 26 and 28 Lm^−2^h^−1^, respectively). Reprinted with permission from Ref. [63]. Copyright 2014 Elsevier.

Figure 7 depicts the flux drop in two trials, and four unique phases were found based on the flux decline pattern. The patterns of flux change were the same in both experiments (E1 for the first experiment and E2 for the second experiment). The flux decreased dramatically over the first six days (phase I: first–sixth day) from roughly 30 to 6 Lm^−2^h^−1^ and then stabilized at 2 and 4 Lm^−2^h^−1^ for the remaining 30 days of operation (phase IV) until the end of E1 and E2, respectively. The flux variation exists in a similar pattern observed in a previous GDM system study reported by others [49].

### 5.4. Fouling Layer Development

**Figure 8:**
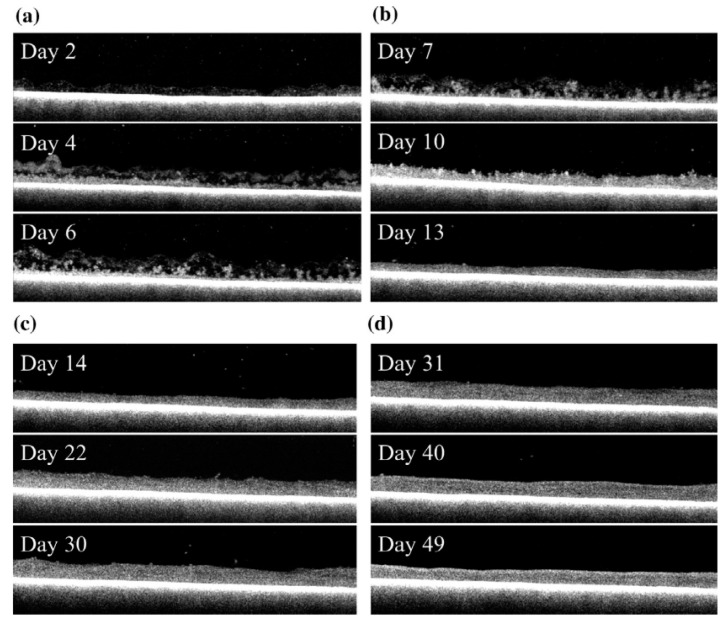
Evolution of fouling morphology by optical coherence tomography scanning: (**a**) phase I, (**b**) phase II, (**c**) phase III, and (**d**) phase IV. Reprinted with permission from Ref. [62]. Copyright 2014 Elsevier.

Wang et al. [62] used optical coherence tomography scanning to determine the morphology of the fouling layer, which evolved from a thin, loose, and porous structure to a thick, dense, and homogeneous structure in both tests (Figure 8). In general, the change in morphology corresponded closely to the change in flux and resistance, which may also be classified into four phases.

#### 5.4.1. Phase 1

Under the dead-end filtration condition, the non-dissolved and colloidal material in the feed was retained and accumulated on the membrane, including bacteria, organic aggregated colloidal material, and particulate organic and inorganic material. Organic compounds maintained on the membrane might promote bacterial growth [49]. As shown in Figure 8a, the pore is plugged, and biofilms with reduced permeability initially had a thin and fluffy structure. However, by the end of phase I (day 6), a smooth and compact layer was discovered in the bottom section of the biofilm, where the flux had decreased to a substantially low level (Figure 8). In phase I, the thickness rose linearly during the first several days as extra fouling resistance developed, and the flux decreased dramatically.

#### 5.4.2. Phases 2 and 3

Beginning on day 7, when phase II (Figure 8b) began and a brief period of steady flow was observed, the fouling layer was exposed to the deposition of materials of various sizes, including bacteria, colloids, and particulate organic and inorganic compounds [64]. Following that, as illustrated in Figure 8, with foulants aggregating and biofilm compacting, the thickness of the fouling layer decreased at the end of phase II after a brief fluctuating period due to the fouling layer becoming a more homogeneous structure. The dynamic equilibrium between heterogeneity and thickness can explain the temporary flux stabilization. However, the biological process was more active, and the fouling structure was more diverse at the same time. As a result, the increased resistance may be compensated for, resulting in steady flux.

During phase III (Figure 8c), the measured thickness of biofilm increased progressively due to continual filtration. Meanwhile, more organic compounds such as biopolymers were trapped in the fouling layer, facilitating bacterial development and increasing biopolymer excretion. These gel-like substances filled the void and constricted the pores. As a result, the fouling layer became denser, increasing fouling resistance and decreasing flow.

#### 5.4.3. Phase 4

Accumulation will halt when the flux of solids into the system equalizes the flux of solids out. The degree of flow stabilization was temperature and hydrostatic pressure-dependent [56]. However, increased hydrostatic pressure or lower temperature increased fouling resistance, reducing the flow. After entering phase IV, the biofilm was compacted into a uniform shape, and thickness fluctuated within a narrow range. Additionally, the relative roughness coefficient remained fairly low, indicating a robust and uniform biofilm structure. Additionally, it is noted that the fouling thickness significantly decreased throughout this time. This is most likely related to the disintegration of an aged biofilm and the condensation of accumulated biomass.

## 6. GDM Performance

GDM systems have been used in decentralized potable water treatment, decentralized non-potable water treatment, wastewater treatment, and pretreatment of saltwater for desalination. As seen in Table 1, the stabilized flux levels in GDM systems are linked to the feed water type. In general, feed water with a larger concentration of organic compounds forms a more resistant biofilm, resulting in permeate flux in the following sequence: wastewater < rainwater < lake water.

According to Table 2, L. Truttmann et al. [63] revealed that a membrane with a smaller pore size resulted in a 3× increase in the stable flux, which could also be caused by the difference in the membrane material. This is further proven by comparing X. Du et al.’s [65] and A. Ding et al.’s [57] reports, where the UF membrane with a smaller pore size achieved a higher stable flux. However, it should be emphasized that the pressure difference is ignored since, as proved in several studies, modifying the TMP did not result in a significant change in the stable flux value in the GDM system [10,58,60]. This suggests that when the TMP applied to the membrane grew during filtration, the total resistance of the fouled membrane increased as well. This effect was attributed to the fouling layer being squeezed at higher pressures, resulting in lower porosity and increased resistance.

Table 3 shows that GDM effectively removes pollutants from various types of feed. However, L. Truttmann et al.’s [66] experiment demonstrated that it has a detrimental effect on lakes, increasing the conductivity, dissolved oxygen, biopolymers, and building blocks, which could be attributed to the low degree of foulant compared to green algae-polluted water, which GDM is effective on. In addition, GDM has been particularly effective in removing dissolved organic carbon and turbidity [66,67,68,69].

## 7. Confounding Effects of Compaction, Wetting, and Fouling and Future Research Outlook

As previously stated, wetness, fouling, and compaction significantly influence the ULPM filtration system. Those three phenomena can occur simultaneously, particularly during the earlier stage of filtration. If each of them is not taken into consideration, the outcome of the measurement might be misleading. The permeability value might differ depending on the testing pressure and duration (level of compaction) and the level of prewetting. For example, in a recent study, increasing the testing pressure from 2.5 to 19 kPa reduced the clean water permeability by a factor of five in the ULPM system [10], which was in line with other studies reported by Zainuddin et al. [26]. In those studies, the membrane was prewetted will ethanol as a low-surface-tension liquid. Therefore, those data might become irrelevant for a full-scale system operated under different conditions. In order to avoid such a mismatch, it is recommended to evaluate the permeability under the expected operational TMP.

The wettability of the membrane is also very important. Since most commercial membranes for pressure-driven membrane filtration are hydrophilic, immersion in water will wet the membrane pores. However, due to the hydrophobic nature of the main polymer, the supporting structure (the polymer matrix) might be dry and hydrophobic and left dry for module assembly and module transportation prior to installation. In order to avoid pore collapse, an impregnating agent such as glycerol is often used [70,71]. Hence, a pore activation protocol is normally applied, such as prewetting with ethanol, acetone, and isopropyl alcohol [21]. For instance, ethanol pretreatment could increase the hydraulic performance of a polysulfone UF membrane by three folds [22]. Such a simple protocol is still hard to implement on a household decentralized system scale, leading to performance loss. Therefore, more accessible pore activation methods are highly encouraged by employing material available locally. Detergent solution with a low concentration (around 0.05–0.1%) is one of the alternatives recommended for pore activation of membranes used in ULPM filtration, which is also beneficial for cleaning when required [66,72].

Another alternative to combat performance loss due to compaction and pore deactivation is through membrane developments specifically targeting the formation of a structure resistant to deformation even under ULP. Due to the emergence of the ULPM filtration system, this approach is not yet reported in the literature for this purpose but has long been recognized in developing RO membranes. Typically, the membrane support used in RO should consist of minimum macrovoids to withstand the high pressure required for seawater desalination using RO [67].

The GDM concept introduced by Eawag (https://www.eawag.ch/en/ accessed on 19 April 2022) was designed to operate over a prolonged operation without membrane cleaning. Such requirements may be strictly required for certain designs and limited resources to perform the cleaning according to a certain protocol. When the system is designed to allow simple cleaning, the ULPM filtration system can be accompanied by a simple cleaning protocol. For example, the Skyjuice membrane module design (https://skyjuice.org.au/ accessed on 19 April 2022) allows a simple physical cleaning protocol to restore performance. On a household scale, a compact membrane system can easily be cleaned by mechanical hand-shaking [68,73] or rinsed with a detergent solution (https://www.ultrafiltration.gdpfilter.co.id accessed on 19 April 2022). Like a rotary system that allows module rotation [74], innovative module systems are also practical in small-scale ULPM systems to allow a certain degree of membrane cleaning. Overall, the concept of a membrane system without cleaning can still be revisited to enhance the system throughput but without ignoring the niche of robust systems, which can operate without electricity but still be adaptive to available resources to enhance the system throughput.

The key factor determining the application potential of ULPMF is the stable flux value, which greatly determines the treatment cost. Most analyses focused on membrane fouling or the foulant layer on the membrane surface in recent works. Increasing the stable flux remains the main obstacle to the widespread acceptance of ULPMF in large-scale implementations. The high stable flux values (10–20 Lm^−2^h^−1^) are attributed to the low organic content and predator activity that enhances the biofilm porosity. Conversely, the feed contains substances with high membrane fouling propensity (i.e., greywater), leading to low, stable flux values (<5 Lm^−2^h^−1^) [55].

The generic approach seems to improve biofilm quality favorable for low resistance (i.e., heterogeneous and low density) or minimize biofilm through pretreatment. The former seems challenging and hard to control when operating small-scale and possibly unsupervised systems. The latter seems more promising, especially via the extended biofilm volume in the attached growth system. When some potential foulant can be largely removed in the pretreatment stage, membrane properties are expected to be more prominent because the cake/biofilm resistance is lower than or comparable to the intrinsic membrane resistance. Additionally, it might remove eukaryotes, which are known to perform roles in forming heterogeneous biofouling structures. At this point, it is not very clear how the biofilm can be managed. Increasing the eukaryote activity by increasing temperature seems impractical, as demonstrated elsewhere [75]. On the other hand, increasing the surface area for biofilm by introducing a carrier or even altering the filtration configuration seems more practical [76,77].

The most important consideration for ULPMF implementation is the total cost (CAPEX + OPEX). It is more favorable than the traditional UF for low- or medium-scale installation [25]. Despite the few available cost data in the literature, it highly depends on the site condition. Therefore, individual assessment per site is required to judge the economy of the process. For large-scale applications, a collaborative study with a commercial partner is required to truly judge the viability of ULPMF in comparison to traditional UF.

Many recent reports also explored the application of ultra-low pressure in different applications. The studies were motivated by the nature of fouling, which is highly reversible. ULPMF was reported to be applied in anaerobic palm oil sludge filtration with substantially low energy input [78]. The introduction of bubbling compensated for the high solids in the feed. ULPMF was also explored for simultaneous recovery of detergent and water from laundry wastewater [68,73]. The advantage of low energy in ULPMF is highly attractive to enhance the net energy recovery of processing microalgae biomass into biodiesel by lowering the energy input in biomass harvesting [10].

In summary, ULPM filtration systems display exceptional efficacy in removing pollutants. They may play a critical role in increasing worldwide access to drinking water. However, the fundamental issues of membrane compaction, membrane wetting, and membrane fouling can still be addressed to enhance the performance.

## Figures and Tables

**Table 1:** Overview of membrane filtration performance reported for gravity-driven membrane systems fed with different types of water.

Water Source	Stable Flux (Lm^−2^h^−1^)	Membrane Pore Size	Membrane Type	Applied Pressure (mbar)	Total Hydraulic Resistance (×10^12^ m^−1^)	Refs.
Lake water	~2.54	0.1 μm	MF	75	12–13	[63]
	~6.55	100 kDa	UF	75	3.6	
Green algae-polluted lake water	~1.45	0.1 μm	MF	75	20.0	
	~9.42	100 kDa	UF	75	2.6	
Rainwater	~4.0	0.1 μm	MF	400	-	[65]
	~4.5	150 kDa	UF	50	5.0	[57]
Wastewater	~0.18	-	UF	70	-	[58]
River water	~2.0	0.22 μm	MF	70	-	[59]
	~3.9	150 kDa	UF	65	6.0×10	[60]

**Table 2:** Impact of membrane properties on the membrane performance in gravity-driven membrane filtration.

Materials	Pore Size	Membrane Area (m^2^)	Stable Flux (Lm^−2^h^−1^), Pressure (mbar)	Refs.
PSU	100 kDa	-	~90, 75	[63]
PVDF	0.1 μm	0.207	~27, 75	
	0.1 μm	0.1	~4.0, 400	[65]
	-	0.001	~0.18, 70	[58]
	0.22 μm	0.4	~2.0, 70	[59]
PES	150 kDa	0.031	~3.9, 65	[60]
	150 kDa	0.00125	~4.5, 50	[57]

**Table 3:** Removal efficiency for organic compounds by gravity-driven membrane systems in treating different water matrices.

Compounds	Removal in GDM (%)	Filtration Time (day)	Feed Water	Refs.
Biopolymers	−15.6	34	Lake water	[63]
	11.5	34	Green algae-polluted lake water	
Building blocks	−17.7	34	Lake water	
	17.5	34	Green algae-polluted lake water	
COD	40.0	160	Rainwater	[65]
DOC	71.4	55		[57]
	57.1	55		[61]
	60.0	120	River water	[59]
	5.0	63		[60]
Turbidity	99.3	160	Rainwater	[65]
	98.6	120	River water	[59]
DO	−2.3	34	Lake water	[63]
	−14.3	34	Green algae-polluted lake water	
Conductivity	−1.9	34	Lake water	
	7.2	34	Green algae-polluted lake water	
TOC	13.2	34	Lake water	
	22.8	34	Green algae-polluted lake water	
	13.2	160	Rainwater	[65]

## Data Availability

Not applicable.
